# Overview of the effects of chemical mixtures with endocrine disrupting activity in the context of real-life risk simulation: An integrative approach (Review)

**DOI:** 10.3892/wasj.2019.17

**Published:** 2019-08-05

**Authors:** DENISA MARGINA, GEORGE MIHAI NIȚULESCU, ANCA UNGURIANU, ROBIN MESNAGE, MARINA GOUMENOU, DIMOSTHENIS A. SARIGIANNIS, MICHAEL ASCHNER, DEMETRIOS A. SPANDIDOS, ELISAVET A. RENIERI, ANTONIO F. HERNÁNDEZ, ARISTIDIS TSATSAKIS

**Affiliations:** 1 ‘Carol Davila’ University of Medicine and Pharmacy, 020956 Bucharest, Romania;; 2 Gene Expression and Therapy Group, Department of Medical and Molecular Genetics, Faculty of Life Sciences and Medicine, King’s College London, London SE1 9RT, United Kingdom;; 3 Department of Forensic Sciences and Toxicology, Faculty of Medicine, University of Crete, 71409 Heraklion;; 4 Department of Chemical Engineering, Environmental Engineering Laboratory, Aristotle University of Thessaloniki, 54124 Thessaloniki;; 5 HERACLES Research Center on the Exposome and Health, Center for Interdisciplinary Research and Innovation, Balkan Center, 57001 Thessaloniki, Greece;; 6 Environmental Health Engineering, Department of Science, Technology and Society, School for Advanced Study (IUSS), 27100 Pavia, Italy;; 7 Department of Molecular Pharmacology, Albert Einstein College of Medicine, Bronx, NY 10463, USA;; 8 Laboratory of Clinical Virology, School of Medicine, University of Crete, 71409 Heraklion, Greece;; 9 Centre of Toxicology Science and Research, School of Medicine, University of Crete, 71409 Heraklion, Greece;; 10 Department of Legal Medicine and Toxicology, University of Granada School of Medicine, Granada, Spain

**Keywords:** real-life risk simulation, chemical mixtures, endocrine disruptors

## Abstract

Research over the past years has indicated that chronic human exposure to very low doses of various chemical species in mixtures and administered via different routes (percutaneous, orally, etc.) should be the main focus of new biochemical and toxicological studies. Humans have daily contact with various chemicals, such as food additives, pesticides from fruits/vegetables, antibiotics (and other veterinary drugs) from meat, different types of preservatives from cosmetics, to name a few. Simultaneous exposure to this wide array of chemicals does not produce immediate effects, but summative effect/s over time that may be clinically manifested several years thereafter. Classical animal studies designed to test the toxic outcome of a single chemical are not suitable to assess, and then extrapolate to humans, the effects of a whole mixture of chemicals. Testing the aftermath of a combination of chemicals, at low doses, around or below the no observed adverse effect is stressed by many toxicologists. Thus, there is a need to reformulate the design of biochemical and toxicological studies in order to perform real-life risk simulation. This review discuss the potential use of computational methods as a complementary tool for *in vitro* and *in vivo* toxicity tests with a high predictive potential that could contribute to reduce animal testing, cost and time, when assessing the effects of chemical combinations. This review focused on the use of these methods to predict the potential endocrine disrupting activity of a mixture of chemicals.

## 1. The need for a change in the current scientific paradigm

**Figure 1.**
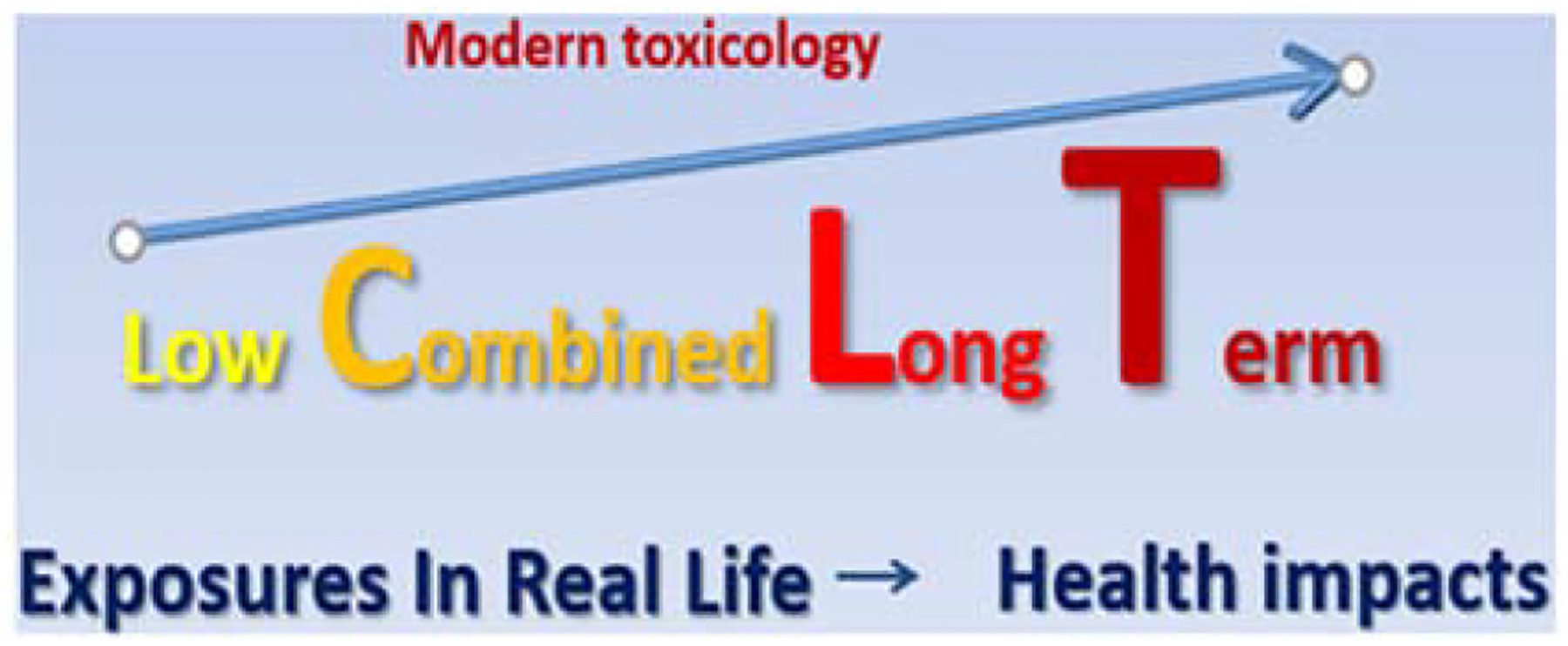
Paradigm change in the toxicological study design.

Human populations come into contact, on a daily basis, with a large range of chemical mixtures, at low levels of exposure, from virtually every product that is used, from the food consumed (raw or processed, either vegetal, containing soil originating substances, or animal), drinking water (tap or bottled), the air that is breathed, consumer products (cosmetics, either artisanal or industrial), etc. The results from a nation-wide survey of environmental contaminants among 4,145 pregnant women in France in 2011 indicated that bisphenol A, phthalates, pesticides (such as pyrethroids), dioxins, furans, polychlorobiphenyls, brominated flame retardants, perfluorinated compounds and heavy metals are quantifiable in virtually all individuals (1). Over the past 15 years, researchers from different disciplines (toxicologists, biochemists, chemists, medical doctors and molecular biologists) have made constant efforts to unravel the possible effects induced by the long-term exposure to low doses of chemicals on human physiology (2) (Fig. 1).

**Figure 2.**
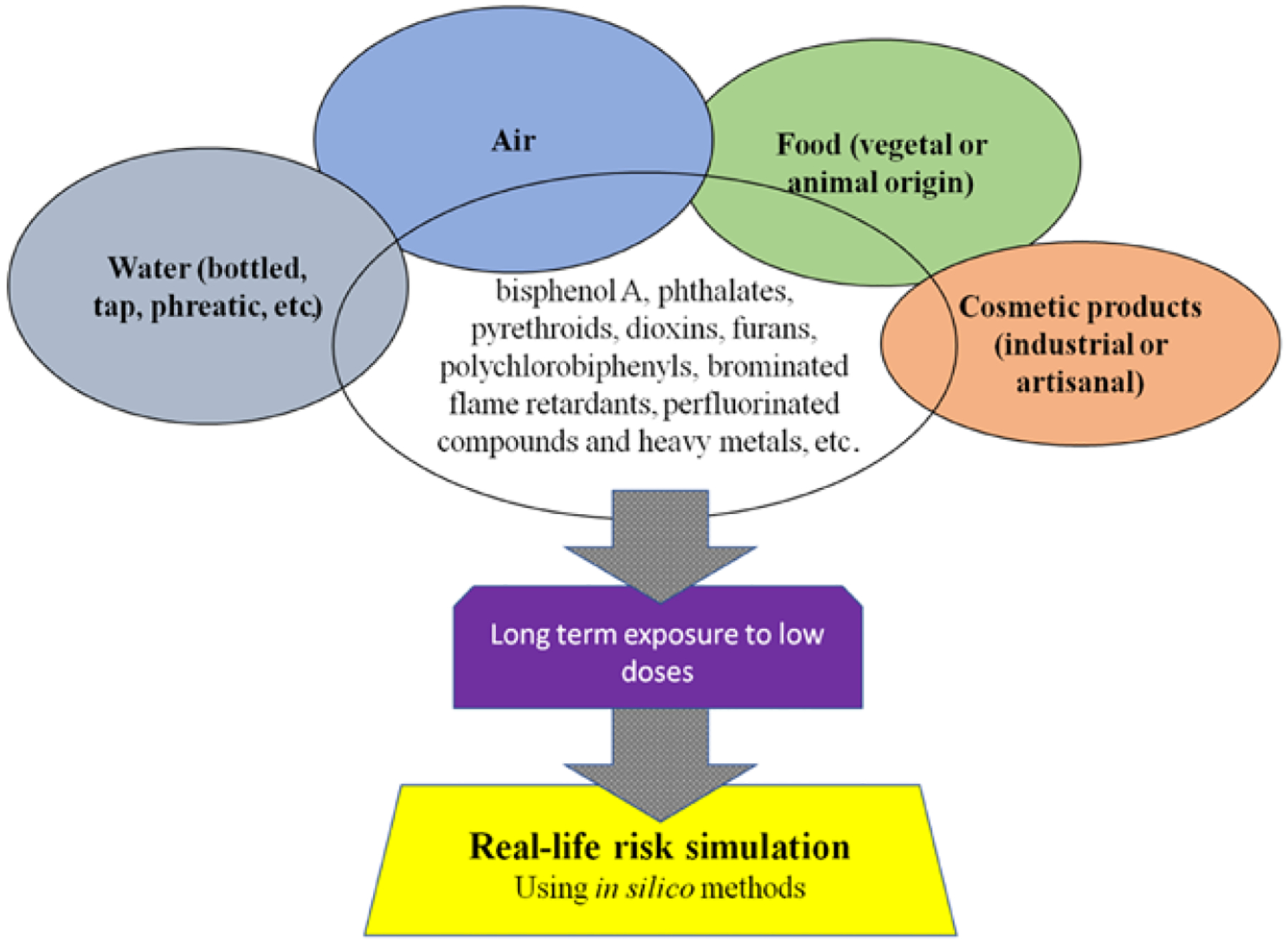
Low-dose chronic exposure to chemical mixtures: An important issue in contemporary studies.

The realization that humans are exposed to a large number of substances through different routes in their everyday lives, has led to a change in scientific paradigms (2). To date, the majority of studies, and in particular regulatory toxicology studies, have focused on testing multiple outcomes resulting from the administration of a single substance at medium-high doses to laboratory animals (3). Also, biomonitoring studies (carried out mostly in urine samples) have confirmed exposure to different chemicals. Although the presence of chemicals (or their metabolites) in biological samples does not constitute a proof of the occurrence of adverse health effects in humans, it represents a source of concern. However, there is growing evidence of the effects of chemical mixtures at concentrations for which individual components failed to elicit adverse effects when tested individually (4,5) (Fig. 2).

Data on the effects of chemical mixtures are increasingly taken into consideration by the scientific community and regulatory agencies worldwide to issue regulations and guidelines to understand better the long-term effects of exposure to environmental (or dietary) mixtures of chemicals in real-life exposure scenarios and to protect/preserve the human health. Although humans are not exposed simultaneously to all existing chemicals; the assessment of an infinite number of potential chemical combinations in not feasible from a regulatory point of view. Hence, the most representative chemical mixtures, and their risk drivers, should be identified at first, and then validated and internationally accepted tools can be applied to assess their potential combined effects.

Toxicological studies testing combinations of chemicals at low doses, around or below their no observed adverse effect level (NOAEL), mimicking real-life scenarios, under the framework of real-life risk simulation (RLRS), are imperative to evaluate the effects induced by these chemical mixtures in humans (6–13).

Another area of research for future studies investigating the effects of mixtures of environmental pollutants is the role of the gut microbiome. Communities of microorganisms inhabiting the human gastrointestinal tract have the ability to metabolize a large range of chemicals and affect their therapeutic efficacy or their toxicity (14). Since the potential chemical metabolism of the gut microbiome remains largely uncharacterized, recent reviews have encouraged the conduct of studies simulating real-life exposure to mixtures using laboratory animals or simulators of the human gut microbiome ecosystem (15). Machine learning algorithms could also help developing reliable approaches to simulate gut microbiome metabolism and its consequence on human health in future studies (16).

## 2. Relevance of exposure to mixtures of endocrine disruptor chemicals in the context of 21st century research

One of the most important areas of concern regards the potential health effects of exposure to low doses of mixtures of endocrine disruptors (EDs) also known as endocrine-disrupting chemicals (EDCs). Modern lifestyles result in ubiquitous daily exposures to a combination of environmental mixtures of EDs that can accumulate in the body tissues and fluids. Human exposure, particularly at very low-doses, is continuous and occurs in different mixtures with potential effects that may not be predictable when evaluating individual compounds. Thus, the assessment of potential human risks resulting from exposure to mixtures of EDs is crucial for consumer safety (17). Moreover, recent evidence indicates that exposure to these chemicals during development can affect not only the exposed individuals, but also their offspring and future generations as a result of epigenetic modifications (18).

Specifically, synthetic compounds can contain polycyclic aromatic structures, resembling the structure of endogenous hormones. By interacting on specific receptors, and depending on their concentration, affinity and potency, they can elicit effects by mimicking natural hormones. For this reason, such chemicals can also exert effects even at very low concentrations (e.g., steroid hormones, such as dehydroepiandrosterone sulphate (DHEAS) can have effects at femtomolar concentrations) (19). By interfering with physiological endogenous systems, EDs impair the hormone balance and disrupt normal function, ultimately inducing toxicological effects. Exposure to such substances is of particular concern in sensitive periods, such as the prenatal period, as these exposures can lead to irreversible changes in the developing organs and increase the susceptibility to develop diseases later in life. Nevertheless, there is still controversy concerning the possible role of exposure at real-life concentrations to environmental chemicals and certain endocrine related human diseases, such as hormone-related cancers, reproductive disorders, obesity, diabetes and neurodevelopment disorders (20). Certainly, EDs interfere with brain development through changes in thyroid hormone levels that are essential for the development of the nervous system.

Different International Organizations and Agencies have provided a similar definition for EDs. WHO defines an ED as ‘an exogenous substance or mixture that alters function(s) of the endocrine system and consequently causes adverse health effects in an intact organism, or its progeny, or (sub)populations’ (21). This is also the working definition adopted by the European Commission (EC) (22). The European Food Safety Authority (EFSA) inserted the term ‘Endocrine Active Substances (EASs)’ defined as ‘any chemical that can interact directly or indirectly with the endocrine system, and subsequently result in an effect on the endocrine system, target organs and tissues’ (23). The reason for inserting this term was to discriminate between chemicals that may interfere with the endocrine or hormone systems without inducing adverse outcomes.

The Environmental Protection Agency (US-EPA) defined EDs as ‘exogenous agents that interfere with the production, release, transport, metabolism, binding, action, or elimination of the natural hormones in the body responsible for the maintenance of homeostasis and the regulation of developmental processes’ (24). EDs have been linked from fertility disturbances to a number of highly prevalent human pathologies, such as obesity, cancer and diabetes mellitus (23–32). Trasande *et al* (2015) estimated that EDs contribute at least €157 billion per year to the cost of human disease in the European Union (EU) (33). In the US, the estimated figure is even larger, reaching $340 billion per annum (34).

There are some points to be considered when discussing EDs in the context of RLRS. Firstly, there is the incredible chemical diversity of EDs. These can include natural substances from plants and/or fungi (such as phytoestrogens), pharmacologically active molecules (such as contraceptive hormones or molecules used in hormone-responsive malignancies), chemicals used as additives, preservatives in food/cosmetics, pesticides, solvents, lubricants, fungicides and other types. Chemical structures also vary considerably, some of them being clustered based on their common structure, such as polychlorinated derivatives, bisphenols, dioxins, phthalates, or diethylstilbestrol (35,36).

Existing assays are currently focused on the estrogen, androgen, thyroid and steroidogenesis (EATS) pathways and less on non-EATS modalities. However, standard chronic apical toxicity tests are capable of detecting most downstream effects of perturbation of the non-EATS pathways (20).

A useful toxicological tool for EDs is the Endocrine Disruptor Knowledge Base (EDKB), an online library available at the US FDA, containing experimental data for >3,200 chemical compounds and serves as a resource for both research and regulatory scientists (37). Based on the EDKB, the National Center for Toxicological Research (NCTR) of the US is currently developing methods and models for the computational prediction of endocrine-related risks.

Similarly, the Endocrine Active Substances Information System (EASIS) was developed in the EU. EASIS can be used to search for results from scientific studies on chemicals related to endocrine activity. Currently, it contains information on >500 different chemicals based on *in vitro* and *in vivo* assays in various species. The presence of a substance in the database does not mean necessarily that it is an ED. A new and improved version, EASIS 2.0, is anticipated to be published soon (38).

The Organization for Economic Cooperation and Development (OECD), in 2018, updated the document entitled ‘Revised Guidance Document 150 on Standardized Test Guidelines for Evaluating Chemicals for Endocrine Disruption’ as a standard for the assay to be used for the identification of new EDs based on endocrine signaling pathways (39). When data are lacking, the document advises the use of quantitative structure-activity relationship (QSAR) models, category, and read-across assessment for hazard identification. On its website, the OECD made available a free QSAR Toolbox that can be used as standalone software or for a better interpretation of the mechanisms underlying *in vivo* results.

The European Commission requested that EFSA and ECHA develop a common harmonized guidance to ensure that the endocrine disruptor criteria adopted by the EU in 2017 are applied consistently for the assessment of biocides and pesticides. For drafting this guidance, the Joint Research Centre (JRC), the EC’s science and knowledge service provided its support due to its expertise in the area and previous reports development (23).

The US Environmental Protection Agency (EPA) has a dedicated Endocrine Disruptor Screening Program (EDSP) to identify substances that have the potential to interact with the estrogen, androgen, or thyroid hormone systems and to establish a dose-effect relationship. The program uses two major exposure models, the first being ‘off-the-shelf’ chemicals released into the environment by the industry, and the second concerns consumer and in-home chemicals ingredients (40). Recent studies have shown that the ToxCast database can be profit-ably used to elucidate the mechanisms of action of chemicals acting as obesogens, such as neonicotinoids (41), or as estrogen receptor agonists, such as bisphenol A alternatives (42).

## 3. Future directions in real-life risk simulations of EDs

EDs mixtures used in experimental studies are very simple and consist of unrealistic mixtures compared to the real-world scenario. As such, the net effect in humans of the mixture of numerous EDs with diverse activities is unpredictable and requires further developments. Computational methods are an essential tool in the drug discovery process, and they are intensively used for the identification of new EDs, considering the time and cost consuming efforts to test all household and industrial chemical ingredients. Computational methods are an important complementary tool for *in vitro* and *in vivo* toxicity tests with a high predictive potential that can contribute to identify and assessing risks, and ultimately to reduce animal testing, cost and time (43). The application of machine learning methods on toxicological ‘big data’ has already been shown to outperform animal test reproducibility (36). This has also been proven to be a successful strategy for determining the effects of chemical mixtures, such as those comprised by EDs. For example, a recent study identified that both the pharmacological estrogen, 17α-ethinylestradiol, and the pesticide, trans-Nonachlor, were not able to activate the pregnane X receptor (PXR) individually; however, when combined, they were efficacious. A biophysical analysis complemented by structural bioinformatics analysis revealed that these compounds formed supramolecular ligands, allowing the combined chemical structure to fit into and activate the ligand binding pocket of the PXR (44).

There is a wide range of computational models, varying from read across, chemical categories, absorption, distribution, metabolism, and elimination (ADME) predictive models, physiologically-based pharmacokinetic (PBPK) models, quantitative structure activity relationships (QSARs), docking and molecular dynamics that are currently used to identify new EDs and to predict their mechanisms of action (45). The predictive power of these methods depends on their selectivity and specificity (46). For example, some studies have suggested that molecular docking methods are not the best choice to evaluate androgen receptor antagonists, while the results of QSAR analyses and molecular dynamics simulations have acceptable sensitivities and specificities (46,47). Kar *et al* developed a QSAR model to evaluate mixtures of perfluoroalkyl substances (PFASs), an important class of endocrine-disrupting pollutants, based on zebrafish embryos development data. The predicted chemicals mixtures displayed a concentration addition pattern suggesting a similar mode of toxic action and non-interaction (48). In the case of estrogen receptors, a large range of methods has been applied in large-scale modeling projects (49). Molecular dynamics simulations have also been used to study the interaction between glyphosate and estrogen receptor alpha (42). It is crucial that researchers and all regulatory agencies understand the drawbacks, limitations and confidence limits of each method. As an example, pharmacophore models work very well for estrogen and androgen receptors, although they are limited in the case of various enzymes that control hormone metabolism (50,51).

There is no single tool available with which to identify all types of potentially active groups; thus, several methods need to be used. The accumulation of biological data from several types of assays on EDs will increase the accuracy of the computational models and will certainly expand their usefulness. In addition, with 48 nuclear hormone receptors (52), many more peptide receptors and an unknown number of signaling pathways as potential targets for these chemicals, the conceivable effects on human biological pathways is massive.

Another important point is the difficulty encountered in the extrapolation of the effects induced by the EDs from an *in vivo* tested dose to a RLRS model, based on the fact that the dose-response curves for such chemicals are under an intense debate (53,54). As an example, for a number of years, the regulation of pesticides has been based on the paradigm that larger doses (above the NOAEL) result in larger effects, i.e., ‘the dose makes the poison’. However, studies published over the last 15 years have demonstrated toxic effects of combinations of chemicals at concentrations lower than the NOAEL that can disrupt biological systems (55), suggesting that this area requires further scrutiny (56). The cumulative risk assessment of chemicals in mixtures should be considered in addition to the evaluation of its individual effects (57). Another area of interest that represents a relevant challenge to human health is the non-monotonic dose-response relationships (NMDR). Under this hypothesis, the effects at low doses cannot be predicted from effects at high doses and, if confirmed, chemical testing would need to be changed to protect human health. One review of 51 studies identified 170 non-monotonic dose-response relationships (58); nevertheless, the majority of data comes from *in vitro* studies.

Evaluating the effects induced by mixtures of chemicals, only considering the ED class, can be very challenging due to the multitude of possible complex combinations of chemicals/chemical classes that humans can come in contact with. Sarigiannis *et al* (2009) proposed a comprehensive framework for addressing this challenge (59). The identification of an effective model to test the above-mentioned effects, is the first step in this type of research. There are, of course, two criteria to be met: to find the mixture of molecules that mimics best real-life situations and to find a way to evaluate the effects induced *in vivo*. Since the doses are low, the realization of chronic feeding studies is preferable, but complex and costly. In this context, molecular modeling may constitute a solid first step in such an endeavor.

It is practically impossible to test all the combinations of EDs, even for binary mixtures. The groundbreaking work of Bliss identified three categories of joint action in mixtures (60). In the first pattern, the combined effect is the sum of the components, their toxic effect being independent, even if the toxicological mechanism is the same or not. It is the simplest case, as the proportions of each component do not alter their combined effect. In the second case, the toxic effect is not independent and it can be greater than that of each constituent in the case of a synergistic action or lower in the case of antagonistic effects. In the particular case of ED mixtures, the synergistic action is the most important and several models have been developed to address this problem (61). However, at low exposure levels (around the NOAEL), synergism or antagonism are considered to be unlikely or toxicologically insignificant.

The generalized concentration addition is a mathematical model that evaluates the interaction between mixtures components using a response function independent of the response functions of each individual constituent. The receptor-oriented approach in cumulative risk assessment changes the paradigm from the traditional source-oriented approach, focusing on the exposure assessment of humans to EDs, coupled with effect assessment considering a time variable exposure.

**Figure 3.**
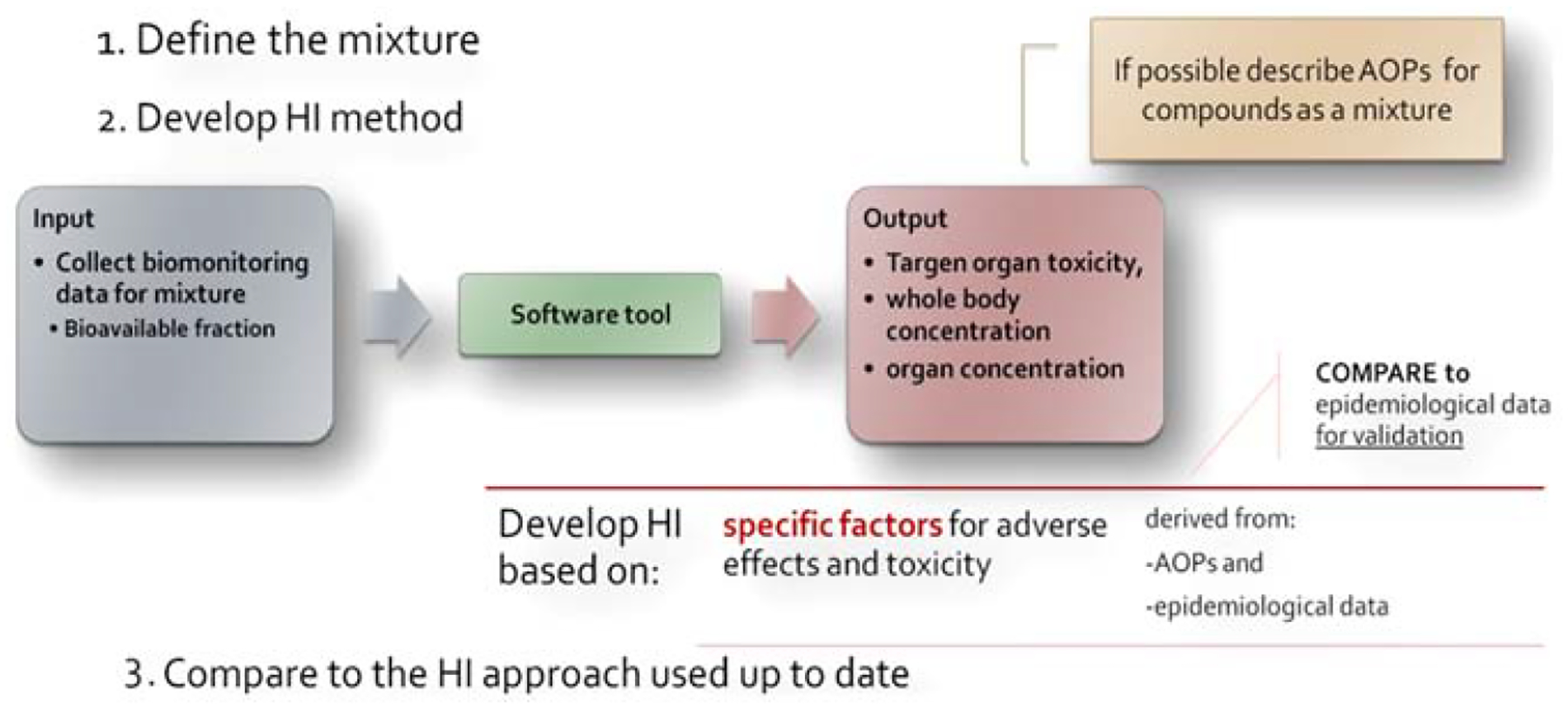
Exposure assessment to real mixtures and HI estimation method: Fit for purpose. HI, hazard index.

Acknowledging the infinite possible combination of mixtures, the development of hazard estimation approaches fit for purpose, instead of the “umbrella” approaches to cover all grounds, might be more appropriate in certain cases (62). With a view to alleviate uncertainties, a method outline for defined mixtures is proposed (Fig. 3). A three-step process is described, where firstly the mixture in question has to be defined. The mixture (components and portions) is meant to reflect real exposures [f.i. measuring the occurrence and concentration of substances in drinking water of a specific area for the development of a specific hazard index (HI)].

Moving to the second step, the basic notion for the development of the HI is to collect human biomonitoring data on the substances determined and via a software tool, which assesses aggregated (e.g., INTEGRA) or cumulative exposure, to obtain data on certain adverse effects’ markers, e.g., biomarkers of target organ toxicity. The data obtained from the in silico model could be integrated with data from other lines of evidence (*in vivo*, *in vitro*, epidemiology) concerning the same compounds. This could help i) fill the gaps in describing AOPs for the specific mixture and not individual substances; and ii) develop an adversity specific HI which is to be compared with the currently applied HI and evaluated accordingly, as the last step of the proposed process. The basic notion is to collect human biomonitoring data on the substances determined and via a software tool, which assesses aggregated (e.g., INTEGRA) or cumulative exposure, to obtain data on certain adverse effects’ markers (target organ toxicity, concentration level, etc). The data obtained from the in silico model could be compared to epidemiological data concerning the same compounds.

**Figure 4.**
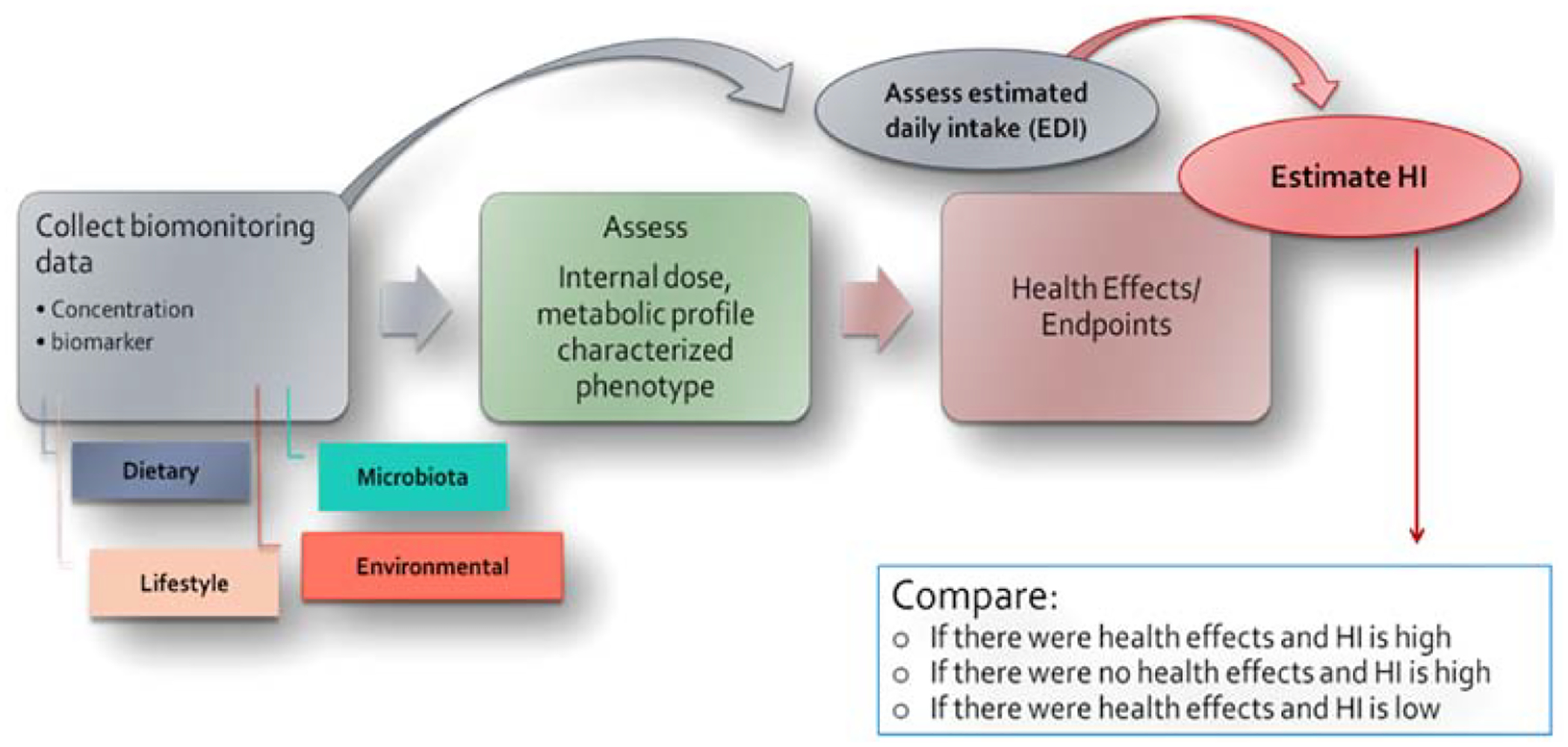
Extensions of the internal dose approach.

Furthermore, aiming to reach more realistic risk characterization methods, an extension of the internal dose approach is proposed (Fig. 4). This approach is based on collecting human biomonitoring data regarding cumulative exposure (dietary, lifestyle, environmental and microbiota) in order to assess the internal dose for the compounds of a specific mixture or characterize the metabolic profile phenotype. Following the internal dose assessment, health effects and toxicity endpoints can be determined. The biomonitoring data could additionally be used for assessing the estimated daily intake (EDI) (63) and subsequently estimating the HI. A comparison between described health effects and the HI estimated could serve as an evaluation of the method (64).

## 4. Conclusions

Endocrine-mediated adverse effects of chemical mixtures cannot be always identified in standard toxicological studies performed to comply with regulatory requirements. Therefore, supplementary and more focused mechanistic studies may be necessary to further investigate an endocrine mode of action. Despite all limitations, it can be considered that the use of in silico methods to evaluate complex RLRS models will have a great impact and such methods will become a powerful toxicological tool. Those methods can contribute to the identification of potential new EDs and to the prediction of their toxicological targets, thus becoming an effective method to concentrate on similar toxicity pathways and mechanisms of action.
